# Cytotoxicological Investigation of the Essential Oil and the Extracts of* Cotula cinerea* and* Salvia verbenaca* from Morocco

**DOI:** 10.1155/2018/7163961

**Published:** 2018-10-14

**Authors:** Fatima-Ezzahrae Guaouguaou, Mohamed Alien Ahl Bebaha, Khalid Taghzouti, Abdelhakim Bouyahya, Youssef Bakri, Nadia Dakka, Nour Eddine Es-Safi

**Affiliations:** ^1^Mohammed V University in Rabat, LPCMIO, Materials Science Center (MSC), Ecole Normale Supérieure, Rabat, Morocco; ^2^Laboratory of Animal Physiology, Faculty of Sciences, Mohammed V University in Rabat, Rabat, Morocco; ^3^Mohammed V University of Rabat, Faculty of Sciences, Department of Biology, Genomic Center of Human Pathologies, Laboratory of Human Pathologies Biology, Rabat, Morocco

## Abstract

The objective of this work was to investigate the cytotoxicological effect of the extracts (hexane, ethyl acetate, and* n*-butanol) of* Cotula cinerea* and* Salvia verbenaca* in addition to the essential oil of* Cotula cinerea*. These plants are widely used in the Moroccan traditional folk medicine. The cytotoxic effect was explored against two cancer cell lines, Vero and RD, using the colorimetric MTT assay. The obtained results showed that the cytotoxicity differed according to the used extract with an efficient effect of* Cotula cinerea* extracts compared to* Salvia verbenaca*. A potent cytotoxicity was thus observed for the* Cotula cinerea *hexane extract which inhibited the growth of RD cell line at the lowest IC_50_ value (57.21±3.43 *µ*g/mL). This was followed by the ethyl acetate extract and the essential oil with moderate effects against RD cell line and showed IC_50_ values of 187.52±6.27 *µ*g/mL and 173.05±4.46 *µ*g/mL, respectively. On the other hand, different results were obtained and* Cotula cinerea *essential oil was the most cytotoxic with the lowest IC_50_ value (72.72±2.18 *µ*g/mL) against Vero cell line. In the same conditions, higher concentrations were needed in the case of* Salvia verbenaca *extracts. The results of this study showed thus that* Cotula cinerea *essential oil and hexane extract showed significant cytotoxic effects against RD and Vero cell lines, respectively, and could be considered as novel source of antitumor agents. This study is expected to be beneficial for clinical and traditional applications for* Cotula cinerea *as a remedy against cancer and opens new perspectives for further investigations on other types of cancer cell lines.

## 1. Introduction

Cancer is a complex disease caused by several factors such as genetic deregulation, environmental influencing agents, and epigenetic modification factors [1, 2]. According to the World Health Organization, incidence and mortality due to cancer are widely increasing in the world [3]. Despite the appreciable advancements and progress made in cancer treatment, it continues to constitute a serious global health problem, especially with the appearance of multidrug resistance cells. To date various bioactive compounds have been tried against cancer. However, and despite all the progress made, the cancer treatment remains a challenging task for both the patients and the medical staff. The current widely used cancer treatments such as surgery, radiotherapy, systemic therapy, and chemotherapy are very expensive and cause several side and adverse effects [4]. Prolonged cancer treatment is generally costly to patients and complicates patient follow-up in hospital. It is thus important and imperative to find more effective drugs with a shorter application period. Exploring alternative sources of new antitumor agents with specific targets is thus imperative and of great and crucial importance.

Traditional forms of medicine, especially the herbal products deployed for centuries in many parts of the world, could be good alternative for scientific investigation due to their attributes against tumor cell lines. Indeed, plants are rich sources of new compounds from which some secondary metabolites have been shown to possess anticancer properties [5–7]. Furthermore, natural products could be as efficient as synthetic ones with no or less toxicity and are less expensive than synthetic drugs. Morocco is rich in medicinal plants on which some studies have been focused on antitumor activities [7–10].

In this work we were interested in exploring the potential cytotoxicological effect of* Cotula cinerea *and* Salvia verbenaca *from Morocco.* Cotula cinerea *which is of the Asteraceae family is a xerophytic plant widely distributed in sandy and desert grounds [11]. Popularly known as “Gartoufa,” it is widely used in Moroccan traditional medicine to fight against several illnesses such as stomachic disorders and gastrointestinal problems [12].* Salvia verbenaca* is a Mediterranean plant currently belonging to the Lamiaceae family. The plant is widely distributed in Morocco and is widely used in Moroccan traditional folk medicine as a cholagogue, antiseptic, diuretic, and astringent [12]. However, and despite the widespread use of these plants in Moroccan traditional folk medicine, to the best of our knowledge, no information has been previously reported on the anticancer activity of Moroccan* Cotula cinerea *and* Salvia verbenaca. *This work was therefore initiated in order to evaluate the in* vitro* antiproliferative effect of* Cotula cinerea* and* Salvia verbenaca* extracts and essential oil against tumor RD and Vero cell lines.

## 2. Material and Methods

### 2.1. Plant Material

The aerial parts of* Cotula cinerea* were collected in March 2014 in the south of Morocco from the region of Dakhla-Oued Ed-Dahab (province of Aousserd). Those of* Salvia verbenaca* were collected on March 2015 at the locality called Ain Aouda, in the Rabat region, Morocco. The plants were identified by Professor Fennane from the Scientific Institute, Mohammed V University in Rabat. Voucher specimens of both plants were deposited in the herbarium of the Botany Department of this institution. The collected aerial parts were left under shade at room temperature until complete drying.

### 2.2. Extraction

The dried aerial parts of* Cotula cinerea* and* Salvia verbenaca* were crushed and 200 g of each plant material was macerated and stirred for 24 hours at room temperature in an ethanol/water (70:30, v/v) mixture with frequent stirring. The maceration process was repeated twice during 24 h each in order to achieve an exhaustive extraction. After filtration and evaporation of ethanol, the obtained aqueous solution was successively fractionated with hexane, ethyl acetate, and* n*-butanol. Then, solvents of the different obtained extracts were evaporated and the dried residues were used to explore their cytotoxic effect. The hexane, ethyl acetate, and* n*-butanol extraction yields were, respectively, 1.00, 3.00, and 4.50% for* Cotula cinerea* and 1.58, 4.64, and 5.28 for* Salvia verbenaca.*

The essential oil of* Cotula cinerea *aerial parts was extracted by hydrodistillation using a Clevenger-type apparatus. The extraction was achieved during six hours and the obtained oil (yield 0.92%) was stored in a glass vial until use.

### 2.3. Cancer Cell Lines and Culture Medium

The two tumor tested cell lines used in this study were RD (Human Embryonal Rhabdomyosarcoma cancerous cell lines: ATCC N°CCL-136) and Vero (Monkey kidney cancerous cell lines: ATCC N°CCL-81). These two species were obtained from the National Institute of Health, Rabat, Morocco, and were cultivated as previously described by Aneb et al., 2016 [7]. Briefly, cells were grown as monolayers in Minimum Essential Medium (MEM) supplemented with 10% heat-inactivated fetal calf serum and 1% Penicillin-Streptomycin mixture. Peripheral Blood Mononuclear Cells (PBMC) isolated and purified from human blood were used as positive control. These cells were cultivated in RPMI supplemented with 10% heat-inactivated fetal calf serum and 1% Penicillin-Streptomycin mixture. Cultures were maintained at 37°C in 5% CO_2_ and 100% relative humidity atmosphere.

### 2.4. Evaluation of the Cytotoxicity

Before evaluating the antiproliferative activity, the cellular density of each species was determined using light microscopy. When cellular density reached a subconfluence threshold, cells were attached by trypsin-EDTA, washed twice with phosphate buffered saline (PBS), and centrifuged at 150 rpm for 15 min (4°C) and were then suspended in fresh culture medium (MEM). The enumeration of cells was made using Malassez cell count. Before treatment with extracts, 100 *μ*L medium MEM containing 4.10^6^ cells/mL were placed in each well containing MEM and incubated at 37°C in 5% CO_2_/humidified air for 24 h. After 24 h incubation, cells were then treated with the essential oil of* Cotula cinerea* or its extracts (hexane, ethyl acetate, and* n*-butanol) in addition to those of* Salvia verbenaca*. Briefly, 100 *μ*L of each extract at concentrations ranging from 31.25 to 500 *µ*g/mL was dissolved in DMSO (1%) and was added to each well in triplicate. The microplates were then incubated for 48 h at 37°C in air condition of 5% CO_2_. Sterile PBS and 1% DMSO (vehicle) were used as negative controls.

The evaluation of cell viability has been achieved through the use of MTT 3-(4,5-dimethylthiazol-2,5-diphenyl tetrazolium bromide) assay. The test is based on the biotransformation of MTT into formazan crystals by mitochondrial dehydrogenases of living cells. The absorbance of the formazan will then be determined by spectrophotometry at 550 nm. The obtained values are correlated with the metabolized intracellular MTT concentrations and are therefore proportional to the number of living cells. Briefly, 20 *µ*L of MTT (5 mg/mL) was added to each microwell and incubated for 3 hours at 37°C in 5% CO_2_. Tetrazolium salts were cleaved to formazan and the reaction was stopped by addition of 100 *µ*L of 50% (v/v) isopropanol-10% (w/v) sodium dodecyl sulfate (SDS) mixture to each well in order to dissolve insoluble formazan formed after tetrazolium dye reduction [7]. After 30 minutes of incubation at room temperature, absorbance was measured at 560 nm using an ELISA plate reader. Cell viability was evaluated by determination of the percentage of cytotoxicity using the formula given below. The concentrations that inhibit half of the cell population (IC_50_) were obtained by modeling the percentage of cytotoxicity versus concentration of extracts. Experimental data were achieved in triplicate and the obtained results were expressed as means ± SD.

Cytotoxicity (%) = 100 × (absorbance of untreated cells − absorbance of treated cells)/absorbance of untreated cells.

## 3. Results and Discussion

In an ongoing program aimed at the exploration of the phytochemical compounds and the biological activities of medicinal plants of Morocco, we were interested, in this work, in the cytotoxic effect of* Cotula cinerea *and* Salvia verbenaca*. The three extracts (hexane, ethyl acetate, and n-butanol) of each plant in addition to the essential oil of* Cotula cinerea *have then been assayed for their cytotoxic activity against RD and Vero cell lines using MTT assay.

**Figure 1:**
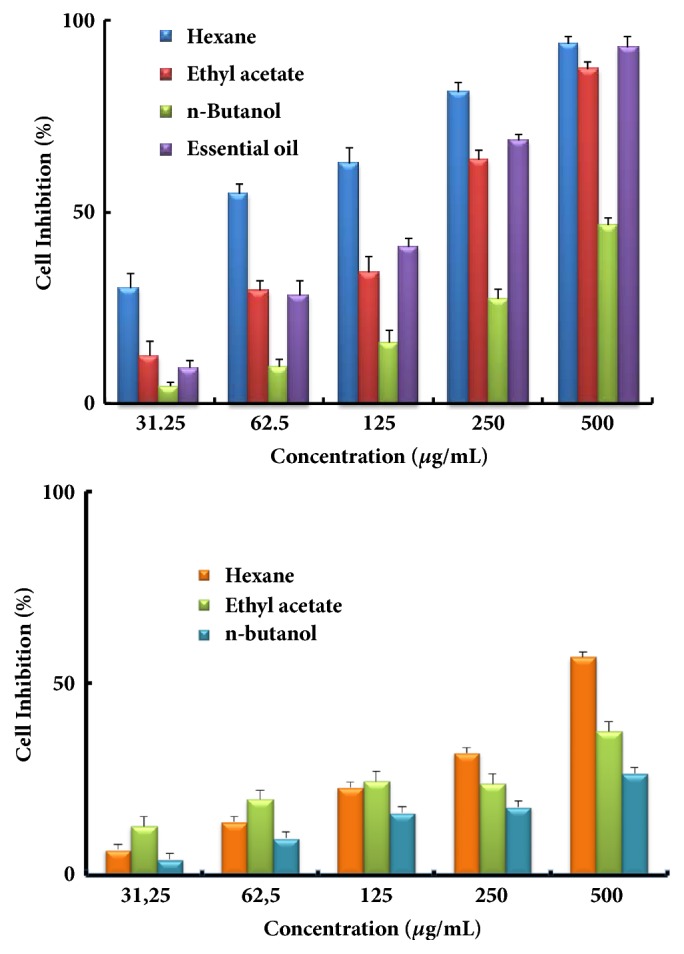
Antiproliferative activity at different concentrations of the essential oil and different extracts of* Cotula cinerea* (top) and* Salvia verbenaca* (bottom) against RD cell line by MTT assay. Results are expressed as means ± standard deviation of three determinations.

**Figure 2:**
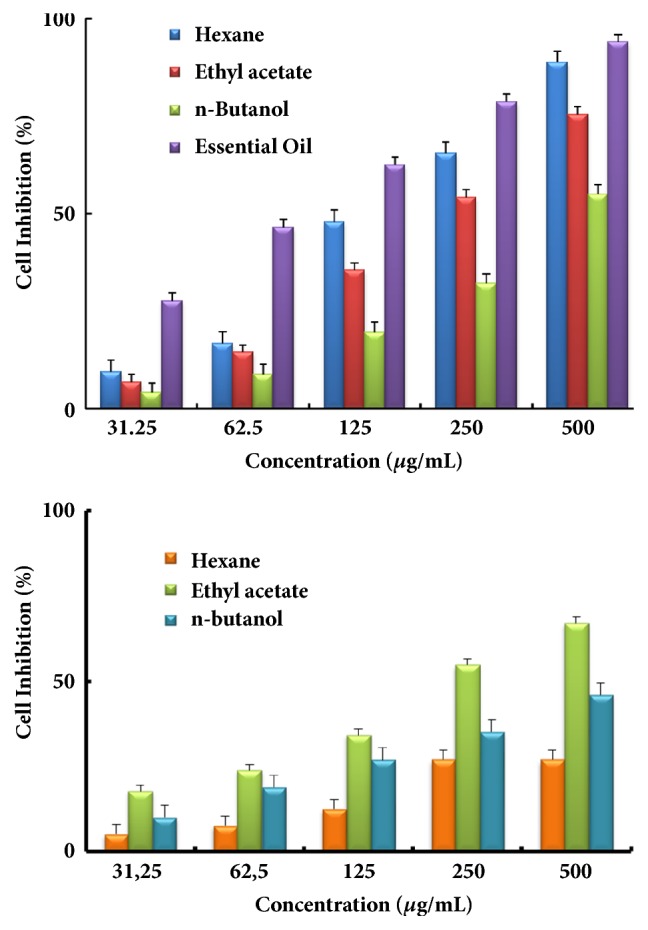
Antiproliferative activity at different concentrations of the essential oil and different extracts of* Cotula cinerea* (top) and* Salvia verbenaca* (bottom) against Vero cell line by MTT assay. Results are expressed as means ± standard deviation of three determinations.

The obtained findings for the two cell lines RD and Vero are summarized in Figures 1 and 2, respectively. The results are expressed as percentage of cytotoxicity versus the concentrations of the essential oil or extracts of both plants.

For both lines, an increase of the cytotoxicity with the concentration of the essential oil or the used extracts was observed indicating a dose dependent effect. An examination of the obtained results showed that the cytotoxic effects of the investigated plants extracts were different according to the tested cell line with an efficient activity of* Cotula cinerea *compared to* Salvia verbenaca*. Comparison of the obtained results for both cell lines showed that* Cotula cinerea* extract activity was higher than its corresponding* Salvia verbenaca *one. This was observed for both cell lines.

In the case of the RD cell line, the* Cotula cinerea* hexane extract showed the higher cytotoxic effect followed by the ethyl acetate, the essential oil, and the* n*-butanol extract. In the same conditions and for the Vero cell line, the best cytotoxic effect was observed with the essential oil followed by the hexane, the ethyl acetate, and the* n*-butanol extracts.

The cytotoxic activity was also expressed in terms of concentration required to inhibit 50% of the tested tumor cell lines (IC_50_ in *µ*g/mL) and the obtained results are gathered in Table 1. In the case the RD cell line, the lowest IC_50_ value (57.21±3.43 *µ*g/mL) was observed with the* Cotula cinerea* hexane extract indicating an important antiproliferative effect. The essential oil and the ethyl acetate extract showed moderate cytotoxic effects (173.05±4.46 and 187.52±6.27 *µ*g/mL, respectively). Higher IC_50_ values (greater than 500 *µ*g/mL) were observed in the case of* Cotula cinerea n*-butanol and most of the* Salvia verbenaca* extracts which presented then the weakest cytotoxicity activity.

In the case of the Vero tumor cell line, Table 1 reveals that* Cotula cinerea* essential oil showed the highest cytotoxic capacity with the lowest IC_50_ value (72.72±2.18 *µ*g/mL) followed by the hexane, the ethyl acetate, and the* n*-butanol extracts (142.27±11.33, 212.83±9.02, and 447.38±6.52 *µ*g/mL, respectively). Higher concentrations were needed in the case of* Salvia verbenaca *extracts which showed the weakest cytotoxic effects.

The noticed differences in the observed cytotoxicity of the investigated mixtures could obviously be due to the phytochemical composition of each extract. The exact mechanism through which the explored extracts act on each tumor cell line is obviously difficult to highlight. Indeed, the explored extracts are complex mixtures containing several phytochemical compounds which could have different biological activities. In addition, these mixtures could act in a synergic or in an antagonism manner.

A preliminary phytochemical screening of the* Cotula cinerea* and* Salvia verbenaca* extracts showed the presence of polyphenols such as flavonoids and other secondary metabolites. These compounds could explain the cytotoxic effect of this extract on RD tumor cell line. Indeed, the antitumor properties of phenolic compounds and flavonoids against several cell lines representing numerous human cell lines have been previously reported [6]. The exact mechanisms through which such compounds act in their cytotoxicity effect are not well known, but some tentative investigations suggested that these compounds could interrupt the life cycle cell, inducing the apoptosis pathways and inactivating the telomerase [6].

The antiproliferative activity of* Cotula cinerea* essential oil against the Vero tumor cell line could be due to presence of cytotoxic molecules in the oil. Essential oils are complex molecules containing various and several terpenoids compounds belonging mainly to monoterpene and sesquiterpene chemical groups and presenting different functional groups such as alcohols, phenols, ketone, etc. [13]. These compounds could act individually or in a synergic manner. The activity of an essential oil is generally attributed and influenced by the major compounds. However, the minor compounds could also be of crucial importance; all the phytochemicals could synergistically act and enhance the observed activity [14]. Moreover, a molecule could have an effect on one type of tumor and not on others.

The action and mechanism of* Cotula cinerea* essential oil is very complex involving several targeting pathways as previously reported [5]. Due to its complex phytochemical composition, it is difficult to define a unique mechanism of its action on the used cell tumor. Nevertheless, the* Cotula cinerea* essential oil can act through various mechanisms. Among these are the activation of the apoptosis via the caspases' family proteins expressions, the modifications in signaling pathways, the interruption of the life cell cycle, the modification in DNA methylation, and histones acetylation of tumor cell lines [5, 15]. The tentative global mechanism gathered probably all these actions which are thus supposed to provide the observed cytotoxic effect. Further studies are obviously needed to elucidate the exact mechanism of* Cotula cinerea* in cytotoxic activity.

## 4. Conclusion

Our results indicated that* Cotula cinerea *essential oil and hexane extract showed significant and encouraging cytotoxic effects against RD and Vero cell lines, respectively, and could then be considered as source of novel antitumor agents. The obtained results raise then the possibility of a potential use of* Cotula cinerea *as a source of potent anticancer products. This study is expected thus to be beneficial for clinical and traditional applications for* Cotula cinerea *as a remedy against cancer and opens new perspectives for further exploration on other types of cancer cell lines. Further investigations regarding the identification of bio-actives molecules responsible for these effects are needed. Examination of the cytotoxic activity of the* Cotula cinerea* individual compounds would give crucial information regarding the exact mechanism of this plant in antitumor activity.

## Figures and Tables

**Table 1:** IC_50_ (*µ*g/mL) values obtained through the cytotoxicity of the essential oil and the extracts of *Cotula cinerea* and *Salvia verbenaca * against RD and Vero cell lines by MTT assay.

**Plants**	**Extracts**	**RD cell line**	**Vero cell line**
*Cotula cinerea*	Hexane	57.21±3.43	142.27±11.33
	Ethyl acetate	187.52±6.27	212.83±9.02
	n-Butanol	>500	447.38±6.52
	Essential oil	173.05±4.46	72.72±2.18

*Salvia verbenaca*	Hexane	474.62±1,31	>500
	Ethyl acetate	>500	223.63±1,61
	n-Butanol	>500	>500

## Data Availability

The data used to support the findings of this study are included within the article.
